# Association of Low Magnesium Level With Duration and Severity of Type 2 Diabetes

**DOI:** 10.7759/cureus.15279

**Published:** 2021-05-27

**Authors:** Ruchir Paladiya, Aakanksha Pitliya, Ayesha A Choudhry, Deepak Kumar, Safana Ismail, Mohammed Abbas, Sidra Naz, Besham Kumar, Amna Jamil, Aliya Fatima

**Affiliations:** 1 Internal Medicine, B. J. Medical College, Ahmedabad, IND; 2 Internal Medicine, Sri Aurobindo Institute of Medical Science, Indore, IND; 3 Medicine, Fatima Jinnah Medical University, Lahore, PAK; 4 Internal Medicine, Jinnah Sindh Medical University, Karachi, PAK; 5 Internal Medicine, Dow University of Health Sciences, Karachi, PAK; 6 Medicine, University of Louisville, Louisville, USA; 7 Internal Medicine, University of Health Sciences (UHS), Lahore, PAK; 8 Internal Medicine, Jinnah Postgraduate Medical Centre, Karachi, PAK

**Keywords:** type 2 diabetes, magnesium levels, association, diabetic control, electrolyte disturbance

## Abstract

Introduction: Type 2 diabetes mellitus can give rise to several complications in the body, including electrolyte imbalance. In this study, we aim to find the association of hypomagnesemia with the duration and severity of diabetes. Understanding the association between magnesium and diabetes may assist in the early detection of hypomagnesemia and help manage the complications associated with electrolyte imbalance.

Methods: This cross-sectional study was conducted in the internal medicine department of a tertiary care hospital in Pakistan from January to March 2021. Three hundred (n = 300) patients with a confirmed diagnosis of type 2 diabetes were enrolled in the study after informed consent via consecutive convenient non-probability sampling. Three hundred (n = 300) patients were included in the study as a reference group. Blood was drawn via phlebotomy and sent to the laboratory to assess glycated hemoglobin (HbA1c) and magnesium levels.

Results: In uncontrolled diabetic patients, mean magnesium level was significantly lower as compared to diabetic patients with good glycemic control (1.34 ± 0.3 mg/dL vs. 1.81 ± 0.5; p-value: <0.0001). Prevalence of hypomagnesemia was significantly more in patients with uncontrolled diabetes, compared to the controlled diabetic group (65.8% vs. 50.8%; p-value: 0.009). In patients with a duration of diabetes of more than 10 years, the mean magnesium level was significantly lower, compared to patients with less than 10 years of diabetes (1.32 ± 0.3 mg/dL vs. 1.78 ± 0.5; p-value: <0.0001). Prevalence of hypomagnesemia was significantly more in patients with diabetes for more than 10 years (64.7% vs. 51.9%; p-value: 0.02).

Conclusion: Hypomagnesemia is prevalent in diabetes and is directly related to the severity and duration of diabetes. It is important to include electrolyte screening as a part of routine screening in diabetic patients for early detection and management of electrolyte imbalance, including hypomagnesemia.

## Introduction

Diabetes mellitus (DM) is a group of disorders that affect the utilization of blood glucose in the body. Hence, leads to excess sugar in the blood, which in turn causes multiple complications [1]. Depending upon the mechanism, there are different types of diabetes, the most common of which is type 2 DM [1]. Among the adult population of Pakistan, there is a trend of the increasing prevalence of diabetes, with it being 13.7% (95% CI, 10.7-17.3) in 2020. The study also showed a higher prevalence in males as compared to the females (13.1% vs. 12.4%), and also a higher incidence in urban than in rural population (15.1% vs. 1.6%) [2].

Type 2 DM can give rise to several complications in the body due to a series of mechanisms, accelerated atherogenesis, insulin resistance, hyperglycemia, and low-grade inflammation. Some of the more serious complications include cardio-cerebrovascular diseases like stroke and heart failure, nephropathy, diabetic retinopathy, diabetic foot, some kinds of cancers, cognitive decline, sleep apnea syndrome, mood disorders, bone metabolism impairments, and electrolyte imbalance, including hypomagnesemia [3].

Magnesium (Mg) is a trace element in our body, which is very important for many physiological processes in either its ionized form or its biologically active form, Mg2+. Depletion of Mg in the body (hypomagnesemia) affects various processes. It inhibits glucose transporter type 4 translocation, induces oxidative stress on endothelial cells, increases insulin resistance, and affects lipid metabolism. Hypomagnesemia can contribute to the initiation as well as the progression of DM [4]. Studies have shown that type 2 DM is associated with an alteration in the levels of Mg in the body, with hypomagnesemia being most prevalent, especially in patients with poorly controlled and chronic diabetes [5]. There is very limited data studying the association between Mg level and the duration and severity of diabetes. In this study, we aim to find the association of Mg level with the duration and severity of diabetes. Understanding the association between Mg and diabetes may assist in the early detection of hypomagnesemia and help manage the complications associated with electrolyte imbalance.

## Materials and methods

This comparative cross-sectional study was conducted in the internal medicine department of a tertiary care hospital in Pakistan from January to March 2021. Three hundred (n = 300) patients with a confirmed diagnosis of type 2 DM were enrolled in the study after obtaining informed consent, via consecutive convenient non-probability sampling. Three hundred (n = 300) patients were included in the study as a reference group. Ethical board approval was taken before enrollment of the patients began. Patients with chronic kidney and gastrointestinal diseases were excluded from the study.

Participants’ detailed history, including age, gender, comorbidities, and duration of diabetes (where applicable) was noted in a self-structured questionnaire. After the history taking, blood was drawn via phlebotomy and sent to the laboratory to assess glycated hemoglobin (HbA1c) and Mg level. Good glycemic control was defined as HbA1c less than 7% [6]. Hypomagnesemia was defined as a serum Mg level of less than 1.8 mg/dL [7].

Statistical analysis was done using the Statistical Package for Social Sciences® software version 23.0 (SPSS; IBM Corp., Armonk, NY, USA). Continuous variables were presented as a mean and standard deviation; whereas, categorical variables as percentages and frequencies. Dependent t-test and chi-square were used as appropriate. A p-value of less than 0.05 represented a difference between the interventional and placebo group, and the null hypothesis was void.

## Results

The age, gender, and smoking status were comparable between both groups. However, the diabetic group had more patients with hypertension, a body mass index (BMI) of more than 25 kg/m^2^, and hypercholesterolemia (Table 1).

**Table 1:** Comparison of demographics and comorbidities BMI: body mass index

Characteristics	Diabetic group (n = 300)	Non-diabetic group (n = 300)	P-value
Mean ± SD (age in years)	51 ± 11	50 ± 11	0.26
Male (%)	189 (63.0%)	192 (64.0%)	0.79
Smoker (%)	112 (37.3%)	121 (40.3%)	0.45
Hypertension (%)	151 (50.3%)	100 (33.3%)	<0.0001
BMI more than 25 kg/m^2^	102 (0.34%)	67 (22.3%)	<0.0001
Hypercholesterolemia (%)	141 (47.0%)	81 (27.0%)	<0.0001

In the diabetic group, 129 participants had uncontrolled diabetes and 119 participants had diabetes for more than 10 years (Table 2).

**Table 2:** Diabetic status of the participants

Characteristics	Frequency
Glycemic Status	
Controlled (HbA1c less than 7.0%)	171 (57.0%)
Uncontrolled (HbA1c more than 7.0%)	129 (43.0%)
Duration of Diabetes	
Less than 10 years	181 (60.3%)
More than 10 years	119 (39.7%)

In diabetic patients, mean Mg level was significantly lower as compared to non-diabetic patients (1.61 ± 0.4 mg/dL vs. 2.04 ± 0.7; p-value <0.0001). Prevalence of hypomagnesemia was significantly more in patients with diabetes as compared to the non-diabetic group (57.3% vs. 19.3%; p-value <0.0001) (Table 3).

**Table 3:** Correlation of magnesium status with diabetic and non-diabetic group

Magnesium status	Diabetic group (n = 300)	Non-diabetic group (n = 300)	P-value
Mean magnesium level (mg/dL)	1.61 ± 0.4	2.04 ± 0.7	<0.0001
Hypomagnesemia (%)	172 (57.3%)	58 (19.3%)	<0.00001

In uncontrolled diabetic patients, mean Mg level was significantly lower as compared to the diabetic patients with good glycemic control (1.34 ± 0.3 mg/dL vs. 1.81 ± 0.5; p-value <0.0001). Prevalence of hypomagnesemia was significantly more in patients with uncontrolled diabetes as compared to the controlled diabetic group (65.8% vs. 50.8%; p-value: 0.009) (Table 4).

**Table 4:** Correlation of magnesium status with and without glycemic control

Magnesium status	Diabetic group with glycemic control (n = 171)	Diabetic group without glycemic control (n = 129)	P-value
Mean magnesium level (mg/dL)	1.81 ± 0.5	1.34 ± 0 .3	<0.0001
Hypomagnesemia (%)	87 (50.8%)	85 (65.8%)	0.009

In patients with duration of diabetes of more than 10 years, the mean Mg level was significantly lower as compared to the patients with less than 10 years of diabetes (1.32 ± 0.3 mg/dL vs. 1.78 ± 0.5; p-value < 0.0001). Prevalence of hypomagnesemia was significantly more in patients with diabetes for more than 10 years (64.7% vs. 51.9%; p-value: 0.02) (Table 5).

**Table 5:** Correlation of magnesium status with duration of diabetes

Magnesium status	Duration of diabetes less than 10 years (n = 181)	Duration of diabetes more than 10 years (n = 119)	P-value
Mean magnesium level (mg/dL)	1.78 ± 0.5	1.32 ± 0 .3	<0.0001
Hypomagnesemia (%)	94 (51.9%)	77 (64.7%)	0.02

## Discussion

The results of our study indicate that hypertension, hypercholesterolemia, and BMI more than 25% were more common in patients with diabetes. A comparison of mean Mg levels between diabetic and non-diabetic patients demonstrated that the prevalence of hypomagnesemia was more common in diabetic patients. Moreover, it was found to be more common in uncontrolled diabetic patients as compared to the controlled ones. The results of our study also showed a major impact of duration of diabetes on Mg levels, highlighting the fact that hypomagnesemia was more common in patients with diabetes for more than 10 years.

The results of our study are in line with other studies, confirming the association of low serum Mg levels and type 2 DM. The study conducted by Winzer et al. highlighted that the prevalence of hypomagnesemia was more common in patients with type 2 DM [8]. Insulin resistance and deficiency inhibits Mg absorption and enhances its excretion through the kidneys [9]. Low Mg levels further decrease the sensitivity of insulin, hence the function of its receptors [10]. The study conducted by Liotta et al. found that low Mg levels were associated with intracerebral hemorrhage, hematoma growth, and worst functional outcomes. Moreover, it has been shown that Mg plays an important role in the coagulation pathways, supporting that hypomagnesemia may have implications in intracranial aneurysm rupture [11]. Sekiya et al. showed that Mg ions have an important role in the functions of factor IX [12]. Moreover, another important complication of hypomagnesemia is acidosis, due to increasing intracellular brain Mg levels and decreasing serum Mg levels [13,14]. According to Agrawal et al., hypomagnesemia further oxidizes low-density lipoprotein, which further promotes atherosclerosis and diabetic macrovascular complications [15]. In addition to this, Sales et al. found that the concentration of Mg in erythrocytes was associated with diabetic neuropathy [9]. Another study conducted by Guerrero-Romero et al. indicated that age, alcohol consumption, and the use of diuretics were major risk factors for low serum Mg levels in diabetic patients [16].

The rationale of our study is to highlight the association between low Mg levels and type 2 DM. Hypomagnesemia is a frequent problem in patients with DM. It majorly affects both glycemic regulation and the occurrence of complications. Moreover, its prevalence increases with the duration of the disease. Also, poor glycemic regulation affects serum Mg levels. We further aim to pay more attention to diabetic patients with low serum Mg levels, but still in the normal range. Moreover, to avoid further complications, electrolyte screening should be a part of the management plan for diabetes. Limitations of our study included that since it was a cross-sectional study, groups were not compared for the development of complications. Secondly, since it was a single-center study, care should be taken while inferring the result to bigger sample size.

## Conclusions

Hypomagnesemia is prevalent in diabetes and is directly related to the severity and duration of diabetes. It is important to include electrolyte screening as a part of routine screening in diabetic patients for early detection and management of electrolyte imbalance, including hypomagnesemia. This can help prevent or reduce the severity of complications.
